# Supplementary material for: Zebrafish Model to Study Angiotensin II-Mediated Pathophysiology
Source: Biology (Basel). 2021 Nov 13;10(11):1177. doi: 10.3390/biology10111177 (PMC8614710; doi:10.3390/biology10111177)
Supplement: Supplementary file 1 [file biology-10-01177-s001.zip › biology-1382475-supplementary.pdf]

Table S1: List of primers used for quantitative RT-PCR analyses.

No.	gene name	Accession Number	Forward Primer	Reverse Primer
1	*fn1a*	ENSDARG00000019815	GGTCATCGTCCAGTCCAG	ATCCACTGAATATGGGTGTT
2	*fn1b*	ENSDARG00000006526	TGGAAATGTGATGCTATTGA	GGCCAATCTGGTAGAACACC
3	*spp1*	ENSDARG00000044276	AAGAAGACGTCTGCCTATGAT	CGCTCATTTGCCTCGAT
4	*tnc*	ENSDARG00000021948	AATGTGTGTGTAACATCGGGTT	ATCACATATGCACATTCCATT
5	*ccn2a*	ENSDARG00000042934	GGTGTACCGCAGTGGAGAGT	CTACAGCACCGTCCAGACAC
6	*loxa*	ENSDARG00000003259	TGAAAGAGCGCAACTTAGCCC	CGTGCGCAAATGCGTAAATG
7	*col1a1a*	ENSDARG00000012405	TATTGGTGGTCAGCGTGGTA	TCCTGGAGTACCCTCACGAC
8	*col1a2*	ENSDARG00000020007	CTGGCATGAAGGGACACAG	GGGGTTCCATTTGATCCAG
9	*col1a1b*	ENSDARG00000035809	GAGGCTACAGAGCCGATGAC	CATTCTGGGTGGCACATCCT
10	*col13a1*	ENSDARG00000045453	TTAGCTGCACGTCTACCGAAG	CCCCCAGGTTTCCCTTTAAGC
11	*col8a1b*	ENSDARG00000003533	AGCCCCGAACCAAATTTCTG	CCCCCGCATGGATATGATGT
12	*col2a1b*	ENSDARG00000011407	ACGGATGCAAGAAACACACAGGA	TCCACAGGGGCAATGTCCACAA
13	*col12a1b*	ENSDARG00000019601	GTGGCTCTCTTTACACTCGC	AAACTTGACAGCGGTTGCTTCCAT
14	*col15a1b*	ENSDARG00000061848	GCGCTTCATGTCATGGAAGA	TAGCCCGTGATGAAGGACAC
15	*myh6*	ENSDARG00000090637	GCAACATGGAGGGCGAGATA	CACTTTGCATTCACCGCCTC
16	*myh7ba*	ENSDARG00000076075	AAGCAGACACTGACCGGAAG	CGTGGCTTGCATGGACTCTA
17	*myh7bb*	ENSDARG00000035322	AAGAGACAAGCTGAGGACGC	CCACGACCTTGACGACATGA
18	*nppa*	ENSDARG00000052960	ATCCTGGGACAGAGACCGAG	CCTATGCGATCCAGCCTTCC
20	*igf*	ENSDARG00000094132	AGAAGGTCACACAACCGTGG	CAGGAAGAGTGGCTATGCCC

Table S2: Mean ct values of quantitative RT-PCR analyses.

Supplementary Table 2A: Mean of ct values used for gene expression analyses respective to Figure 1H.

	igf	*ef1a*
PBS	35.59	18.53
AngII	34.78	15.95

Supplementary Table 2B: Mean of ct values used for gene expression analyses respective to Figure 2D.

	fn1a	*fn1b*	*spp1*	*tnc*	*ccn2a*	*col1a1a*	*col1a2*	*col1a1b*	*col13a1*	*col8a1b*	*ef1a*
PBS	31.01	26.94	32.74	27.63	25.80	24.26	26.30	24.91	34.36	32.79	19.19
AngII	25.90	23.79	31.05	24.54	22.52	20.53	22.01	21.82	30.93	30.67	16.84

	loxa	*col2a1b*	*col12a1b*	*col15a1b*	*ef1a*
PBS	31.465	29.33	29.41	34.33	18.40
AngII	27.9	26.63	29.27	31.31	16.02

Supplementary Table 2C: Mean of ct values used for gene expression analyses respective to Figure 3C.

	nppa	*myh6*	*myh7ba*	*myh7bb*	*ef1a*
PBS	21.78	27.97	26.52	24.80	19.19
AngII	18.31	24.64	23.09	21.90	16.84

Table S3: Gene expression fold changes analyzed by delta-Ct method.

SL#	Gene Name	Fold changes					
		PBS-Control			AngII		
		**Sample1**	**Sample2**	**Sample3**	**Sample1**	**Sample2**	**Sample3**
1	*igf*	1.024	0.988	0.986	0.233	0.587	0.181
2	*fn1a*	0.993	1.011	0.994	5.703	8.931	6.093
3	*fn1b*	0.995	1.011	0.993	1.660	1.815	1.744
4	*spp1*	1.017	0.987	0.994	0.938	0.669	0.398
5	*tnc*	1.065	0.837	1.097	1.469	2.155	1.454
6	*ccn2a*	1.005	1.003	0.990	1.115	2.144	2.195
7	*loxa*	0.996	0.999	1.004	1.884	2.465	2.520
8	*col1a1a*	1.007	0.991	1.001	2.308	2.697	2.827
9	*col1a2*	0.989	1.014	0.996	3.264	4.610	3.756
10	*col1a1b*	0.882	1.063	1.054	1.323	1.867	1.857
11	*col13a1*	0.991	0.988	1.019	1.552	3.079	1.940
12	*col8a1a*	0.978	1.022	0.998	0.556	0.908	1.088
13	*col2a1b*	1.003	1.004	0.991	1.231	1.071	1.465
14	*col12a1b*	1.017	1.005	0.976	0.326	0.188	0.152
15	*col15a1b*	1.008	1.011	0.980	1.203	1.540	2.023
16	*myh6*	0.985	1.026	0.987	1.832	2.048	2.030
17	*myh7ba*	1.009	1.005	0.984	2.017	2.115	2.126
18	*myh7bb*	1.003	1.009	0.987	1.215	1.731	1.272
19	*nppa*	1.009	0.973	1.016	2.039	1.893	2.110
